# A new, unusually large, *Clavicornaltica* Scherer, 1974 flea beetle from Borneo, described and sequenced in the field by citizen scientists (Coleoptera, Chrysomelidae, Galerucinae)

**DOI:** 10.3897/BDJ.12.e119481

**Published:** 2024-03-15

**Authors:** Sean Otani, Luca Bertoli, Filippo Lucchini, Tom P. G. van den Beuken, Desanne Boin, Lehman Ellis, Holm Friedrich, Brittany Jacquot, Sotiris Kountouras, Sarah Yu Rou Lim, Eleonora Nigro, Syafi’ie Su’eif, Wei Harn Tan, Ulmar Grafe, Daniele Cicuzza, Massimo Delledonne, Iva Njunjić, Menno Schilthuizen

**Affiliations:** 1 Tottori University, Tottori, Japan Tottori University Tottori Japan; 2 Taxon Expeditions, Leiden, Netherlands Taxon Expeditions Leiden Netherlands; 3 Mimasaka University, Tsuyama, Japan Mimasaka University Tsuyama Japan; 4 Tottori University of Environmental Studies, Tottori, Japan Tottori University of Environmental Studies Tottori Japan; 5 University of Verona, Verona, Italy University of Verona Verona Italy; 6 Taxon Expeditions B. V., Leiden, Netherlands Taxon Expeditions B. V. Leiden Netherlands; 7 University of Holy Cross, New Orleans, United States of America University of Holy Cross New Orleans United States of America; 8 University of Thessaloniki, Thessaloniki, Greece University of Thessaloniki Thessaloniki Greece; 9 University Brunei Darussalam, Bandar Seri Begawan, Brunei University Brunei Darussalam Bandar Seri Begawan Brunei; 10 University of Copenhagen, Copenhagen, Denmark University of Copenhagen Copenhagen Denmark; 11 Universiti Brunei Darussalam, Bandar Seri Begawan, Brunei Universiti Brunei Darussalam Bandar Seri Begawan Brunei; 12 University of Verona, Verona (VR), Italy University of Verona Verona (VR) Italy; 13 Naturalis Biodiversity Center, Leiden, Netherlands Naturalis Biodiversity Center Leiden Netherlands; 14 MNHN, Paris, France MNHN Paris France; 15 Leiden University, Leiden, Netherlands Leiden University Leiden Netherlands; 16 Taxon Expeditions B.V., Leiden, Netherlands Taxon Expeditions B.V. Leiden Netherlands; 17 Taxon Foundation, Leiden, Netherlands Taxon Foundation Leiden Netherlands

**Keywords:** Lowland Dipterocarp rainforest, citizen science, new species, humicole beetles, taxonomy tourism

## Abstract

**Background:**

The genus *Clavicornaltica* Scherer 1974 consists of very small, soil-dwelling flea beetles in South, Southeast and East Asia. Due to their diminutive size and morphological similarities, very little is known about their ecology and taxonomical diversity. It is likely that further studies will reveal this genus to be much more speciose than the 30 species currently recognised.

**New information:**

A new species of *Clavicornaltica* from Brunei Darussalam is described, *C.mataikanensis* Otani et al., sp. nov. This is the second species of this genus recorded from Ulu Temburong National Park.

## Introduction

Since 2017, the ‘scientific travel agency’ Taxon Expeditions has been organising annual expeditions to field centres in Borneo. During these expeditions, a team of taxonomic experts works together with international citizen scientists and local students to teach a mini-field course in tropical biodiversity and to discover, describe and publish one or more species new to science. The general aim of such ‘taxonomy tourism’ (Schilthuizen 2018) is to make the general public aware of the fact that, even today, the world’s biodiversity is still so incompletely known that it is surprisingly easy to discover new animal species. A habitat that is always a focus on these expeditions is the rich community of micro-arthropods that can be revealed by leaf-litter sieving and Winkler bag extraction. One member of this community is the flea beetle genus *Clavicornaltica* Scherer, 1974, enigmatic because of its cryptic lifestyle.

Whereas most tropical rainforest chrysomelids feed in the canopy or on understorey plants, *Clavicornaltica* species presumably feed on mosses or even leaf litter on the forest floor, a habitat that, until recently and in contrast to specialists of, for example, Staphylinoidea, was rarely investigated by specialists of chrysomelids (Scherer 1974, Damaška and Aston 2019; but see Ruan et al. 2020, Damaška et al. 2021). It is worth noting that they are very small, with most species having a body length of only around 1 mm. These two factors have contributed to the fact that the genus was only discovered in the 1970s. Even with the dozens of species described since then, many believe that this only scratches the surface of their true diversity (Damaška 2019).

Morphologically, *Clavicornaltica* also stands out amongst the Chrysomelidae by having unique clavate-geniculate antennae, which makes them easily recognisable, even to untrained citizen scientists. Not surprisingly, therefore, they have been an easily classifiable element in all leaf-litter sampling and sorting activities on taxon expeditions and we have described two species from previous expeditions: *C.sabahensis* Schilthuizen et al., 2017 from the 2017 expedition to Maliau Basin, Sabah, Malaysian Borneo (Schilthuizen et al. 2017) and *C.belalongensis* Schilthuizen et al., 2019 from the 2018 expedition to Ulu Temburong, Brunei, Borneo (Schilthuizen et al. 2019).

On the 2023 Borneo expedition, also to Ulu Temburong, Brunei, we found a relatively large number of specimens of *Clavicornaltica*, both by Winkler extraction and in-flight interception traps. One of these stood out by its large size (ca. 2 mm), close to the maximum known for the genus. As body size in *Clavicornaltica* appears to vary intraspecifically across only a narrow range (Medvedev 1996, Damaška 2019), this meant that the number of species with which it needed to be compared was limited and led to the realisation that it is a new species, which we here describe.

## Materials and methods

### Fieldwork

**Figure 1.**
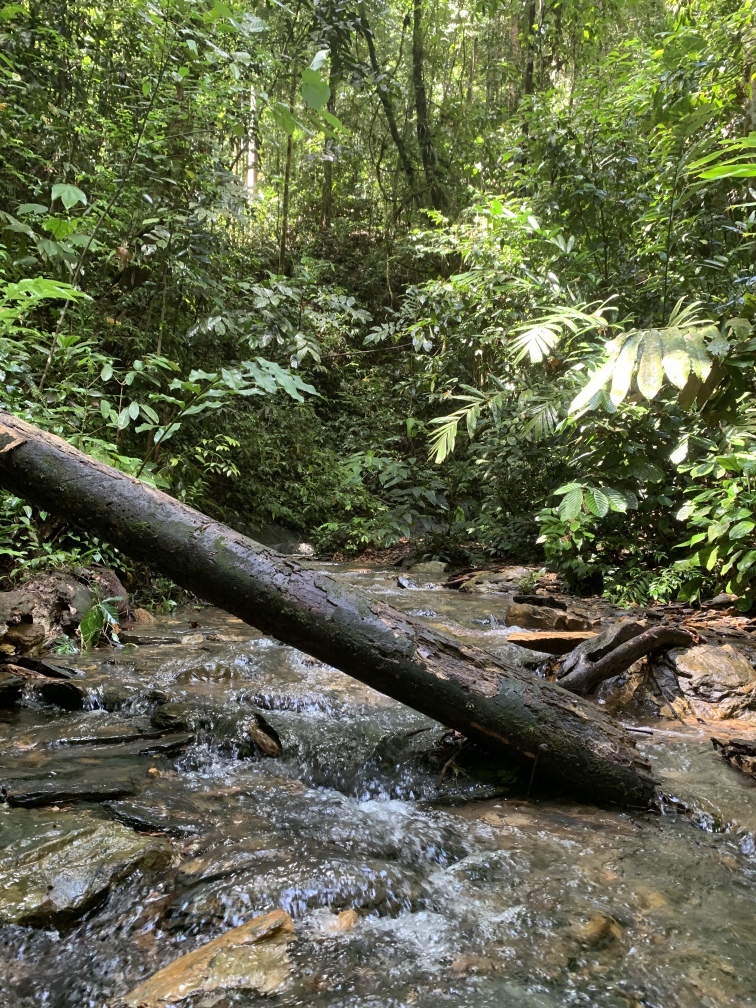
The type locality of *Clavicornalticamataikanensis* sp. nov: the stream bed of the Mata Ikan. The specimens were sieved from leaf litter just off the banks of the stream.

The sampling site was located on the banks of a mountain stream ca. 60 m above sea level (4.5491°N, 115.1561°E) in the Ulu Temburong National Park, Temburong District, Brunei Darussalam (Fig. 1). This location is just a short walk from the Kuala Belalong Field Studies Centre (KBFSC). Sampling was conducted twice. Once on the afternoon of 16 September 2023 and again on the morning of 20 September 2023. Two females were collected during the first sampling and a third female was collected during the second sampling. Leaf litter was collected by hand and sieved on site using standard leaf-litter sieves (manufactured by BioForm, Germany). The sieves had a square mesh size of 1 cm. The flow-through was taken back to KBFSC and placed in Winkler Eclectors (manufactured by Entowinkler, Austria). Collection containers containing a ca. 1 cm layer of 70% denatured ethanol were checked daily.

### Morphology

The following equipment and techniques were used for morphological examination. With a Nikon SMZ445 microscope fitted with 10× eye pieces, we examined morphological features to a maximum magnification of 35×. Photographs were taken with a Sony Alpha-7RII, fitted with two lenses mounted on top of one another: a Laowa 60 mm 2:1 and a Raynox DCR250. A Twin Macro Flash (model: KuangRen KX-800) was used as the light source in the photographs. Images were extracted and stacked in Helicon Focus (Kharkiv, Ukraine). Spermathecae were dissected and photographed in polyvinylpyrrolidone, an embedding medium (Lompe 1986). Measurements of body parts were calibrated with an image of a ruler taken at the same magnification as the specimen.

### Molecular work

Tissue samples from the holotype and one paratype underwent genetic analysis in a portable field laboratory, following the previously established methodology (Menegon et al. 2017, Maestri et al. 2019). DNA extraction from beetle leg tissue involved the excision of small tissue segments. The DNeasy Blood & Tissue kit (Qiagen) was employed, following the recommended protocol. The extracted DNA served as a template for PCR amplification, specifically targeting the cytochrome c oxidase subunit I (COI) barcoding region, using the "Ron" and "Nancy" primers (Simon et al. 1994) tailed at the 5' end with Oxford Nanopore adapters. Subsequently, the COI amplicons underwent electrophoresis on a 0.8% agarose gel to confirm fragment size and assess the absence of contamination. Successful amplicons were uniquely tagged using sample-specific barcodes using the PCR-Barcoding kit (Oxford Nanopore Technologies). To ensure purity, AMPureXP magnetic beads were applied at a 0.5x ratio to remove contaminants. Amplicon quantification was performed using a Qubit fluorometer. A DNA library was prepared from the pool of amplicons with the Ligation Sequencing Kit V14 (Oxford Nanopore Technologies), following the recommended protocol. Sequencing was carried out using the MinION device (Oxford Nanopore Technologies) within an R10.4.1 flowcell, with data acquisition managed by MinKNOW software v. 23.04.3 (Oxford Nanopore Technologies). We only obtained a successful sequence for the holotype.

### Bioinformatics

Following sequencing, the POD5 files generated by the MinION device underwent base-calling using the ONT Preprocessing Pipeline, powered by Guppy 6.5.7, to produce fastq files. These fastq files served as input for the ONTrack2 pipeline (Maestri et al. 2019), which generated a consensus sequence further queried against the NCBI COI database. Due to the absence of *Clavicornaltica* sequences in the NCBI COI database, the newly-generated consensus sequence of the holotype was aligned with a sympatric, congeneric consensus sequence established in an earlier paper (Schilthuizen et al. 2019). The sequence was deposited on the Barcoding of Life Database (BOLD; www.boldsystems.org) under number TXEX079-24.

## Taxon treatments

### Clavicornaltica mataikanensis

Otani, Bertoli, Lucchini, Boin, Ellis, Friedrich, Jacquot, Kountouras, Lim, Nigro, Otani, Syafi’ie, Tan, Grafe, Cicuzza, Njunjić & Schilthuizen sp. nov.

AAE5760D-920B-57BF-B712-2886E154DD6B

Clavicornaltica Scherer, 1974 - Scherer (1974), Medvedev (1996), Konstantinov and Duckett (2005). Type species: *Clavicornalticabesucheti* Scherer, 1974

#### Materials

- **Type status:** Holotype. **Occurrence:** catalogNumber: UBDM.3.06346; recordedBy: Taxon Expeditions field course participants; individualCount: 1; sex: female; lifeStage: adult; preparations: whole animal (dry), card-mounted; disposition: in collection; occurrenceID: B64B1B95-6CD9-5C93-B1E2-329FF04F617A; **Taxon:** kingdom: Animalia; phylum: Arthropoda; class: Insecta; order: Coleoptera; family: Chrysomelidae; genus: Clavicornaltica; specificEpithet: mataikanensis; taxonRank: species; scientificNameAuthorship: Otani et al. 2024; nomenclaturalCode: ICZN; **Location:** continent: Asia; island: Borneo; country: Brunei Darussalam; stateProvince: Temburong; locality: Kuala Belalong Field Studies Centre; verbatimLocality: Ulu Temburong, near Kuala Belalong Field Studies Centre, along Mata Ikan stream; verbatimElevation: 60 m; decimalLatitude: 4.5491; decimalLongitude: 115.1561; **Event:** samplingProtocol: Winkler eclector; samplingEffort: 250 l of leaf litter; eventDate: 16-09-2023; habitat: Lowland dipterocarp forest; **Record Level:** type: PhysicalObject; institutionID: UBD; institutionCode: IBER-UBD; collectionCode: Zoology; basisOfRecord: PreservedSpecimen
- **Type status:** Paratype. **Occurrence:** catalogNumber: UBDM.3.06347; recordedBy: Taxon Expeditions field course participants; individualCount: 1; sex: female; lifeStage: adult; preparations: whole animal (dry), card-mounted; disposition: in collection; occurrenceID: A1593B82-F1B3-5B88-B486-6B82F4A38EDC; **Taxon:** kingdom: Animalia; phylum: Arthropoda; class: Insecta; order: Coleoptera; family: Chrysomelidae; genus: Clavicornaltica; specificEpithet: mataikanensis; taxonRank: species; scientificNameAuthorship: Otani et al. 2024; nomenclaturalCode: ICZN; **Location:** continent: Asia; island: Borneo; country: Brunei Darussalam; stateProvince: Temburong; locality: Kuala Belalong Field Studies Centre; verbatimLocality: Ulu Temburong, near Kuala Belalong Field Studies Centre, along Mata Ikan stream; verbatimElevation: 60 m; decimalLatitude: 4.5491; decimalLongitude: 115.1561; **Event:** samplingProtocol: Winkler eclector; samplingEffort: 250 l of leaf litter; eventDate: 16-09-2023; habitat: Lowland dipterocarp forest; **Record Level:** type: PhysicalObject; institutionID: UBD; institutionCode: IBER-UBD; collectionCode: Zoology; basisOfRecord: PreservedSpecimen
- **Type status:** Paratype. **Occurrence:** catalogNumber: TXEX.COL.01545; recordedBy: Taxon Expeditions field course participants; individualCount: 1; sex: female; lifeStage: adult; preparations: whole animal (dry), card-mounted; disposition: in collection; occurrenceID: 16312EBB-D6F5-5F0D-98E4-C2F72E357A86; **Taxon:** kingdom: Animalia; phylum: Arthropoda; class: Insecta; order: Coleoptera; family: Chrysomelidae; genus: Clavicornaltica; specificEpithet: mataikanensis; taxonRank: species; scientificNameAuthorship: Otani et al. 2024; nomenclaturalCode: ICZN; **Location:** continent: Asia; island: Borneo; country: Brunei Darussalam; stateProvince: Temburong; locality: Kuala Belalong Field Studies Centre; verbatimLocality: Ulu Temburong, near Kuala Belalong Field Studies Centre, along Mata Ikan stream; verbatimElevation: 60 m; decimalLatitude: 4.5491; decimalLongitude: 115.1561; **Event:** samplingProtocol: Winkler eclector; samplingEffort: 250 l of leaf litter; eventDate: 20-09-2023; habitat: Lowland dipterocarp forest; **Record Level:** type: PhysicalObject; institutionID: TXEX; institutionCode: TXEX; basisOfRecord: PreservedSpecimen

#### Description

**Habitus.** Body dark reddish-brown, large, length ca. 2.0 mm, width ca. 1.8 mm, ovoid and convex, nearly hemispherical. Antennae and visible parts of legs yellowish-brown when viewed dorsally. Head slightly lighter than pronotum and elytra.

**Head.** Rectangular, moderately covered in light punctures. Antennae capitate; clava ca. 0.3 mm, posterior surface straight, uncurved; anterior surface convexly curved; clava segment 1 as long as wide, segments 2-4 slightly wider than long, segment 5 longer than wide. Eyes convex, ca. 1/7 the width of the head measured across the eyes in dorsal view, each eye consisting of 30-40 ommatidia.

**Pronotum.** Posterior width ca. 1.1 mm when viewed from above; shiny, slightly duller than the elytra; marginal groove running from the anterior ventral region to ca. 3/5 of the height of the posterior margin, widening posteriorly before narrowing and terminating. Medium to thick scattering of shallow punctures across entire surface. A large setiferous pore ca. 3/4 down the length of the lateral margin with a seta ca. 1.3 times the length of the antennal clava.

**Elytra.** Striae somewhat irregular, the puctures of the lateral striae deepest, becoming very shallow and indistinct on the more dorsal striae. A narrow groove runs along the dorsal side of the lateral margin of the elytra, deepest anterior, dissipating towards the apex.

**Hind wings.** Absent.

**Abdomen.** Majority of abdominal surfaces the same colour as the dorsum, in some parts slightly lighter, pygidium lighter. Upper inside-margin of second abdominal sternite with a series of deep punctures and an acutely raised carina in the shape of an inverted ‘Y’.

**Spermatheca.** Receptacle 0.29 mm long, J-shaped, fairly uniform in width, slightly thinner towards the top; pump indistinct from receptacle; duct uniform in width and U-shaped where it is attached at the top of the receptacle, ca. ¼ the width of the receptacle, then becoming thinner, entire duct ca. ¾ the length of the receptacle, a bulbous attachment at the terminus of the U-shaped section at the point at which the duct becomes thinner. There is some intraspecific variability visible between the two spermathecae that we dissected (Fig. 4): the spermatheca of the paratype is terminally more slender and curved in the holotype than in the paratype.

**Male genitalia.** Unknown

#### Diagnosis

**Figure 2.**
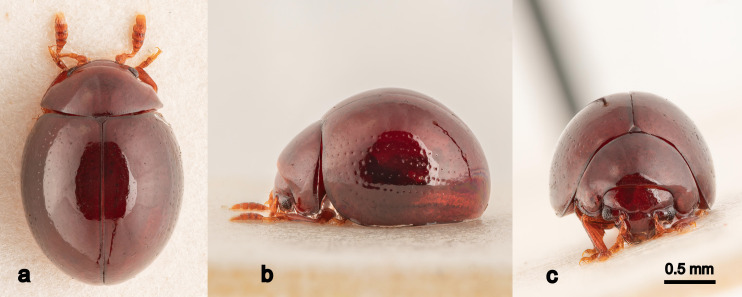
*Clavicornalticamataikanensis* sp. nov., holotype (UBDM.3.06346), habitus in dorsal (a), lateral (b) and frontal (c) views.

**Figure 3.**
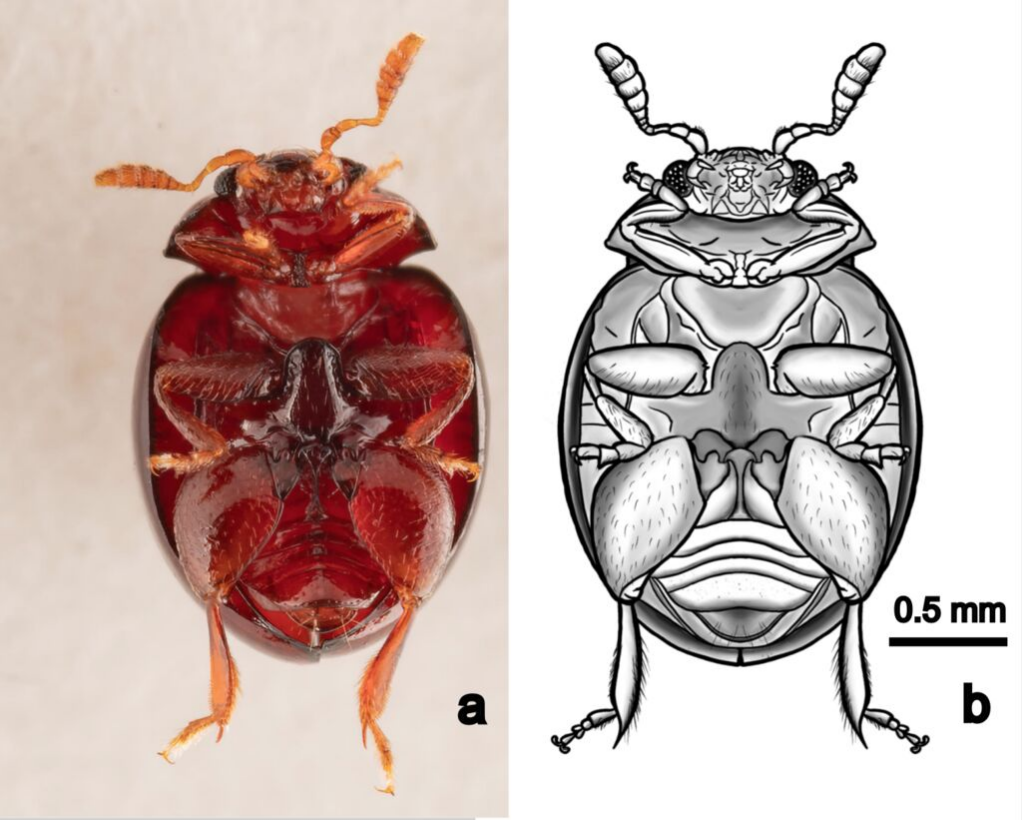
Ventral view (a, photograph; b, drawing) of a paratype of *Clavicornalticamataikanensis* sp. nov. (TXEX.COL.01545).

**Figure 4.**
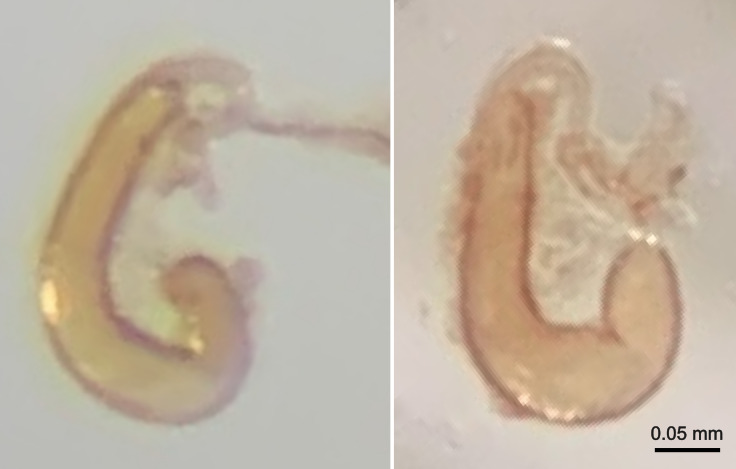
Spermathecae of *Clavicornalticamataikanensis* sp. nov.: On the left, paratype (UBDM.3.06347), on the right, holotype (UBDM.3.06346).

Body dark reddish-brown, large, length ca. 2.0 mm, width ca. 1.8 mm, ovoid and convex, nearly hemispherical. Antennae and visible parts of legs yellowish-brown when viewed dorsally. Head slightly lighter than pronotum and elytra. Eyes ca. 1/7 the width of the head in dorsal view. Scutellum small, triangular. Elytra with punctate rows, deeper laterally becoming shallower dorsally (Figs 2, 3). Spermatheca of characteristic shape (Fig. 4). Male unknown.

##### DNA barcode of holotype (BOLD ID: TXEX079-24)

5'GACTTTCCCTTAGTATATTAATCCGAATCGAATTAAGAAATCCAAGATCATTTATTTCTAATATTCATTTATATAATGTTTTAGTAACAATACATGCTTTTATTATAATTTTTTTTATAATTATACCAATTATAATTGGAGGATTCGGAAATTGATTAATCCCACTAATAATTGGGGCCCCTGATATAGCCTTCCCACGTATAAATAACCTAAGATTCTGATTTTTACCTCCTTCTATAATCTTATTAATTCTTAGTATATTTAGTGAAATAGGAGCAGGAAGAGGATGAACCCTTTATCCCCCATTATCAAATACTTTCTTCCATAATGGACCCGCTATTGACCTAACTATTTTTAGTCTTCATTTAGCTGGAATCTCATCAATCCTTGGAGCAATAAACTTTATTTCTACAATAATTAATATAAAAATTTATAAATTAAAATTTGATCAAATAACCCTCTTTTCTTGAGCTTCCCTTATTACAACTATTCTATTACTATTAGCTTTACCTGTATTAGCAGGAGCTATCACTATACTACTTACAGATCGTAATCTTAATACTTCTTTTTTTGATCCCTCAGGAGGAGGAGACCCCCTATTATAT3'.

#### Etymology

As is customary on our Taxon Expeditions, the name for the new species was decided during a voting session on the last night of the expedition. The proposal which won the most votes was to name it after the stream that runs through the small ravine where the specimens were found, namely Sungai (stream) Mata Ikan. We therefore decided to name it *Clavicornalticamataikanensis* sp. nov. Due to the large number of authors, following Recommendation 51C of the Code (ICZN 1999), the species can be referred to as *Clavicornalticamataikanensis* Otani et al., 2024, provided the full citation of this publication appears in the bibliography or elsewhere in the referring work.

#### Distribution

Known only from the leaf litter on the banks of a small section of the Mata Ikan Stream (Sungai Mata Ikan), between the upper and lower waterfalls where the Ashton Trail crosses the stream (approximately 100 m upstream from where the Mata Ikan Stream enters the Belalong River).

#### Ecology

All specimens were collected on the ground, within several metres on either side of the stream. The Mata Ikan Stream flows through a steep ravine shaded by large trees (e.g. Dipterocarpaceae) with the banks covered in a diversity of saplings, ferns and monocots.

#### Taxon discussion

Differential comparisons were made with all known species of *Clavicornaltica* of similar size and in geographical proximity (geographical distances were calculated in distancefromto.net). *Clavicornalticamataikanensis* sp. nov. can be distinguished from the syntopic *C.belalongensis* Schilthuizen et al., 2019 by the former's larger size and strongly different female genitalia: shorter and pear-shaped (Schilthuizen et al. 2019). *Clavicornalticasabahensis* Schilthuizen et al., 2017, at ca. 230 km distance, is geographically the second closest currently known member of this genus; however, it is distincltly smaller, has more regular elytral striae and a distinctly punctured pronotum. *Clavicornalticairianasarawacensis* Medvedev, 1996 is much smaller (length 1.2 – 1.4 mm) (Medvedev 1996). *Clavicornalticabesucheti* Scherer, 1974 from Sri Lanka is similar in size (holotype: length 1.5 – 2.2 mm; width 1.37 mm), though, with the variability in many of the morphological characteristics described in Forms A-D (Scherer 1974), it is likely that *C.besucheti*, as circumscribed by Scherer (1974), consists of multiple species. It differs from *C.mataikanensis* sp. nov. in having a punctured pronotum and a keel on the first abdominal sternite that is (judging by Fig. 4 in Scherer (1974)) narrow along its entire length and not broadened posteriorly. *Clavicornalticamalayana* Medvedev, 1996, from Peninsular Malaysia is similar in size (length 1.9 mm, width 1.25 mm), has a black as opposed to dark reddish-brown body colour, the raised carina on the second abdominal sternite tapers posteriorly to a point rather than an inverted ‘Y’ and is also separated by considerable geographical distance (ca. 1520 km) (Medvedev 1996). *Clavicornalticabuechei* Medvedev, 2008, from Central Sulawesi is about 25% shorter (length 1.4 mm), has a dark brown to piceous body colour and is geographically distant (ca. 930 km) (Medvedev 2008). *Clavicornalticatarsalis* Medvedev, 1996 from West Papua is smaller, pitch black rather than dark reddish-brown and is geographically separated by ca. 2150 km as well as elevationally (it was collected at 1900 m elevation; Medvedev (1996)). *Clavicornalticatrautneri* Medvedev, 1993 from the Philippines is similar in size (length 2.1 mm), but differs in body colour (Medvedev 1993). *Clavicornalticaphilippinensis* Scherer, 1979, also from the Philippines, is much smaller in size (0.84 – 1.27 mm) (Scherer 1979) and differs in body colour.

## Supplementary Material

XML Treatment for Clavicornaltica mataikanensis
